# Association Between Hyperuricemia, Body Composition, and Comorbidities in an Obese Pediatric Population

**DOI:** 10.1155/jnme/2768062

**Published:** 2025-03-03

**Authors:** João Vasco, Mónica Tavares, Helena Ferreira Mansilha

**Affiliations:** ^1^Pediatric Department, Hospital do Divino Espírito Santo, Ponta Delgada, Azores, Portugal; ^2^Nutrition Unit, Pediatric Department, Centro Materno-Infantil do Norte, ULSSA, Porto, Portugal

**Keywords:** body composition, childhood obesity, MALFD, uric acid

## Abstract

**Background:** Childhood obesity is a global issue with multifactorial causes, leading to chronic and complex disease affecting all organs and systems with high morbidity and mortality, later in life. Elevated serum uric acid (SUA) levels are linked to several comorbidities in adults. In children, however, SUA levels vary by age, sex, and pubertal stage, and therefore, this relation is not well documented. While higher body mass index (BMI) has been associated with SUA levels, other nutritional assessment methods have not been thoroughly investigated and the link between SUA levels and obesity comorbidities in children is underexplored.

**Objective:** This study aims to determine if hyperuricemia is related to body composition and obesity-related comorbidities in children.

**Methods:** A retrospective analysis examined records from 505 obese children aged 5 to 18. The study evaluated the relationship between three nutritional assessment methods: BMI, waist-to-height ratio (WHtR), and body fat mass (BFM) percentage determined by bioimpedance (using InBody 270 scale), SUA levels, and blood markers associated with target organ damage.

**Results:** A significant correlation was found between all three nutritional assessment methods (*p* < 0.001). WHtR showed a stronger correlation with the assessed comorbidities than the BMI z-score. SUA level correlated with HOMA-IR, ALT level, and lipid profile (*p* < 0.001). Multiple linear regression indicated an association between SUA level, BFM percentage, and WHtR (*p* < 0.001). Significant differences in body composition, SUA, and comorbidity markers were observed between children with and without hepatic steatosis (*p* < 0.05).

**Conclusion:** Our results indicated a strong correlation among all nutritional assessment methods; however, WHtR and BFM percentage had a better correlation with obesity complications. SUA is a potential marker for insulin resistance, inflammation, and metabolic dysfunction-associated fatty liver disease (MAFLD) in obese children.

## 1. Introduction

All over the world, obesity in the pediatric population is a public health problem, with a rise in the last decades [1–6]. Although this is an increasing problem, a deceleration in this rise was seen in the last years in developed countries [1]. This plateau, however, was halted by the COVID-19 pandemic, which reinforced the energetic imbalance [7]. In Portugal, between 2008 and 2019, there was a sustained decline in the prevalence of obesity and overweight, in a population of eight-year-old children [8]. However, 2022 COSI report showed a reverse in this trend, following the world tendency, with 32% of the children being overweight, and 14% obese [8].

According to the World Health Organization (WHO), obesity is an abnormal or excessive accumulation of fat that causes harm to one's health [1–3]. It is a chronic and complex disease with a multifactorial etiology with genetic, environmental, and socioeconomic influence [1–3, 9]. In its complexity, it affects all organs, being associated with multiple comorbidities, such as arterial hypertension, glucose metabolism changes, metabolic dysfunction-associated fatty liver disease (MAFLD), sleep apnea, and others [1, 3, 5, 10]. Besides the known consequences in pediatric age, the growing numbers of this disease in such a young age may lead to higher morbidity and mortality later in life [6, 11], bearing a big burden to patients, their families, and the public health system [4, 6, 7].

From the myriad of the already known metabolic and inflammatory changes associated with obesity, such as insulin resistance, hypertriglyceridemia, reduction of high-density lipoprotein (HDL) cholesterol [1, 3, 6, 10], or the elevation in high-sensitive C-reactive protein (hs-CRP) [1, 12], it has been described an association between these changes and the elevation of serum uric acid (SUA) [4, 9, 13–15]. Uric acid is the end product of purine metabolism, and it is produced in the liver and excreted by the kidneys [9, 13, 14]. In physiological concentration, it is known to play a protective role in oxidative stress [9, 13, 16]; however, hyperuricemia has been linked to cardiometabolic disease [4, 9, 13–15] and is an independent risk factor for hypertension, coronary heart disease, and others [16–18]. Furthermore, higher SUA levels can precede the development of metabolic syndrome and are directly related to MAFLD [17, 19, 20]. Although hyperuricemia has been associated with obesity, there are other factors that can be responsible for it, such as dietary intake [14, 21–23] (with purine-rich diets being strongly linked to it), overproduction of uric acid [21–23] (e.g., excessive cell breakdown in neoplastic diseases or genetic disorders), and under-excretion of uric acid occurring in renal diseases [21–23] (being the kidney the main responsible for uric acid excretion and reabsorption) or genetic diseases related to renal or intestinal urate transport deficiency [21–23]. Serum concentration of uric acid increases with age, from the first years of life until puberty, being similar to adulthood at this stage, with differences between sexes [14, 15, 22, 23]. There is no consensus about SUA levels in pediatric age [13–15, 19], but some authors have suggested cutoffs by age and sex [14, 15, 23]. Some of the mechanisms responsible for age-related differences in SUA are increased dietary purine intake, increase in weight, and decreased renal excretion with age [22]. The differences in sex, where boys present with higher SUA levels after puberty, are mainly due to higher lean mass [15] and lower renal excretion [22] when compared with same-age girls.

In the adult population, the association between SUA levels and cardiovascular risk is well established [15, 17, 18], whereas in the pediatric population, in part because of the variability on its levels, this association is not so well documented [15, 24]. In the last few years, some studies carried out in pediatric population have been published, describing an association between the increase of SUA levels and some comorbidities, such as changes in glucose metabolism [5, 13], dyslipidemia [5, 13], MAFLD [19, 20, 25, 26], and others.

In clinical practice, obesity is defined by the body mass index (BMI), using percentile or z-scores published by WHO. However, BMI is not a direct method for the estimation of total body fat mass (BFM) [2, 27, 28]. Waist-to-height ratio (WHtR), although also an indirect method, has been shown to have a better correlation with abdominal and total fat mass [27–30]. Bioimpedance analysis, although being less accurate in the pediatric population than in adults, has shown to have a good correlation with BFM, especially in obese children, although dual-energy X-ray absorptiometry (DEXA) is universally accepted to be the gold standard method for body composition analysis [27, 31].

The association between increased BMI and higher levels of SUA has been described [5, 13]; however, there are no studies evaluating the relationship between SUA and other body composition assessment methods [5, 24]. Our study aims to evaluate if there is an association between levels of SUA, different nutritional assessment methods, and pediatric obesity comorbidities. Additionally, we investigated whether there was a difference in SUA levels and other comorbidities among subjects with hepatic steatosis.

## 2. Materials and Methods

### 2.1. Study Design and Population

This study was designed as a retrospective analysis. It was conducted in a tertiary university Hospital in Porto, Portugal. All subjects were selected from outpatient follow-up at the Nutrition Unit of the Pediatric Department, from January 2021 to December 2023, with a diagnosis of obesity. Patients with monogenic or syndromic obesity, endocrinopathies, or neoplastic diseases were excluded. A total of 505 children and adolescents, between 5 and 18 years old, were included.

A second retrospective analysis was made in the subgroup of subjects which performed abdominal ultrasound (US), a total of 303 patients.

None of the 505 study participants were undergoing cardiovascular therapeutic interventions, although 19 individuals were receiving treatment with metformin.

### 2.2. Data and Body Measurements

Weight and height were measured when participants were wearing only underwear. Height was measured using a wall stadiometer with an accuracy of 0.1 cm. Body weight was measured in the bioimpedance scale (InBody 270) with an accuracy of 0.1 kg. Body composition was assessed according to the InBody manual—measured with no shoes, socks, or heavy clothes; removing all jewelry; and standing on the device barefoot. BFM percentage was measured with InBody 270 scale with an accuracy of 0.1%. Muscular mass (MM) was measured with InBody 270 scale with an accuracy of 0.1 kg. BMI (kg/m^2^) was determined by dividing weight in kilograms by the square of height in meters. Obesity was defined accordingly with the WHO criteria, with a z-score equal or above 2 adjusted for age and sex. Waist circumference was measured, with nonextensible measurement tape, in the middle line between the lowest rib and the iliac crest with an accuracy of 0.1 cm.

In patients with more than one assessment of body measures, the record closest to the date of blood sample collection was considered, and all the other records were discarded.

Digital clinical records were consulted, and the following parameters were obtained: sex, age at evaluation, and Tanner stage.

### 2.3. Laboratory and Radiologic Studies

Uric acid was obtained through uricase method, fasting glucose using hexokinase method, fasting insulin through electrochemiluminescence, alanine and aspartate transaminases using the International Federation of Clinical Chemistry-modified method, total cholesterol and low-density lipoprotein (LDL) were obtained through cholesterol oxidase–peroxidase method, HDL using homogeneous enzymatic colorimetric method, and triglycerides though glycerol-kinase colorimetric method—all using a Roche Cobas C702 equipment. Glycated hemoglobin (HbA1c) was obtained by high-performance liquid chromatography using a Tosoh G11 analyzer. hs-CRP was determined by nephelometry with a Siemens Dimension Vista.

HOMA-IR was calculated by multiplying fasting insulin (μU/mL) with fasting glucose (mg/dL) and dividing the result by 405.

The presence of hepatic steatosis was determined by abdominal US, as it is a noninvasive, low-cost, nonionizing, and widely available imaging technique. Fatty infiltration often manifests on US as increased echogenicity compared with adjacent right kidney or spleen [26]. Abdominal US has a greater sensitivity when fat infiltration is more than 30% of total hepatocytes [26].

### 2.4. Statistical Analysis

Statistical analysis was conducted using IBM SPSS Statistics Version 27. Descriptive statistics were used to characterize the study population. Categorical variables are presented as frequencies and percentages, while continuous variables as means and standard deviations (SDs), or medians and interquartile ranges (IQRs) for variables with skewed distributions. Normal distribution was checked using the Shapiro–Wilk test or skewness and kurtosis, as appropriate.

Categorical variables were compared using Fisher's test or chi-square's test according to their distribution. Continuous variables were compared using Student's t-test or Mann–Whitney as appropriate. Pearson's correlation analysis was used for normally distributed parameters, and Spearman's rho correlation analysis was used for non-normally distributed parameters.

We used multiple linear regression to identify variables that made an important contribution to the variability of SUA and to adjust for confounding variables with analysis of covariance.

## 3. Results

### 3.1. Descriptive Statistics (Table 1)

A total of 505 participants were included in this study, with a mean age of 11.31 ± 3.2 years, of which 261 were boys (51.7%). As stated before, all participants were obese, with a median BMI z-score of 2.77 (IQR_25–75_ 2.46–3.21) and a median WHtR of 0.61 (IQR_25–75_ 0.57–0.64). Concerning body composition, the mean BFM percentage was 42.7 ± 6.2 and the mean MM percentage was 30.7 ± 3.6.

The mean value for SUA was 4.9 ± 1.3 mg/dL. Median fast glucose was 85 (IQR_25–75_ 80–89) mg/dL and median fast insulin was 17.7 mg/dL (IQR_25–75_ 12.0–27.4), with median HOMA-IR of 3.69 (IQR_25–75_ 2.45–5.81). HbA1c presented a median value of 5.3% (IQR_25–75_ 5.1–5.5). The median value of total cholesterol was 152 mg/dL (IQR_25–75_ 136–170), HDL 46 mg/dL (IQR_25–75_ 40–54), LDL 88 mg/dL (IQR_25–75_ 72–103), and triglycerides 83 mg/dL (IQR_25–75_ 58–110). The median value for aminotransferases was 20 U/L (IQR_25–75_ 17–25) for AST, and 18 U/L (IQR_25–75_ 14–24) for ALT. Lastly, hs-CRP revealed a median value of 1.83 mg/L (IQR_25–75_ 0.83–3.96).

Of the 505 participants, a total of 303 (60%) performed an abdominal US and 96 (31.7%) of those revealed hepatic steatosis by this method. An analysis comparing the group with and without hepatic study is present on Supporting Table 1.

### 3.2. Bivariate Correlations (Table 2)

Regarding the BMI z-score, there was a negative low-strength correlation with HDL (*p*=0.001), and a negative moderate correlation with MM percentage (*p* < 0.001). A very low-strength positive correlation was found with AST (*p*=0.024), ALT (*p* < 0.001), fast insulin (*p* < 0.001), HOMA-IR (*p* < 0.001), HbA1c (*p*=0.016), total cholesterol (*p*=0.023), LDL (*p*=0.005), and triglycerides (*p*=0.009). A low-strength positive correlation was found with hs-CRP (< 0.001), and a positive moderate correlation with WHtR (*p* < 0.001) and BFM percentage (*p* < 0.001).

The WHtR showed a very low positive correlation with SUA (*p* < 0.001), ALT (*p* < 0.001), LDL (*p*=0.006), and triglycerides (*p* < 0.001). We found a low positive correlation of WHtR with fast insulin (*p* < 0.001), HOMA-IR (*p* < 0.001), and hs-CRP (*p* < 0.001); and a moderate correlation with BMI *z*-score (*p* < 0.001) and BFM percentage (*p* < 0.001). A very low negative correlation of WHtR with HDL (*p* < 0.001) and a low negative correlation with MM percentage (*p* < 0.001) were shown.

BFM percentage presented a very low-strength positive correlation with total cholesterol (*p*=0.035), LDL (*p*=0.013), triglycerides (*p*=0.008), and HOMA-IR (*p* < 0.001). A low-strength positive correlation between BFM percentage and fast insulin (*p* < 0.001) and hs-CRP (*p* < 0.001), and a moderate-strength correlation with BMI z-score (*p* < 0.001) and WHtR (*p* < 0.001). A very strong negative correlation was seen with MM percentage (*p* < 0.001), and a very low negative correlation with AST (*p* < 0.001).

SUA showed a very low positive correlation with WHtR (*p* < 0.001), MM percentage (*p* < 0.001), and ALT (*p* < 0.001). A low-strength positive correlation with triglycerides (*p* < 0.001), fast insulin (*p* < 0.001), and HOMA-IR (*p* < 0.001), and a moderate positive correlation with age (*p* < 0.001) was found. A low negative correlation was shown between SUA and HDL (*p* < 0.001). There was no statistically significant correlation between SUA and BMI or BFM percentage.

### 3.3. Multivariate Analysis

In the multivariate analysis, our goal was to find if there was a relation between SUA and BFM percentage. Multiple linear regression was used with SUA as dependent variable and age, WHtR, BFM percentage, MM percentage, ALT, triglycerides, and HDL as covariables (Table 3). We found a statistically significant association between SUA and BFM percentage, with an increase of 1% in BFM leading to an increment of 0.118 mg/dL in SUA (*p* < 0.001), adjusted to the variables mentioned before. There was also an association between SUA and WHtR (*p* < 0.001).

### 3.4. Hepatic Study Analysis

In this analysis, we compared the groups that were submitted to US, comparing the one with and the one without hepatic steatosis, and found a statistically significant difference between both with BMI z-score, WHtR, BFM percentage, SUA, ALT, triglycerides, hs-CRP, fast insulin, and HOMA-IR being higher in the steatosis group (*p* < 0.001, Table 1). In the steatosis group, the median of HDL is also lower (*p* < 0.001, Table 1). All the other variables (*p* > 0.05) are represented in Table 1. The bivariate correlations in the group with steatosis are represented in the Supporting Table 2.

## 4. Discussion

In this study, we aimed to understand whether hyperuricemia was somewhat linked to body composition changes and if there was a relationship with obesity-related comorbidities, such as insulin resistance, dyslipidemia, hepatic abnormalities, or inflammation.

There was a moderate positive statistically significant correlation between all three methods of body assessment, which shows that the less-used WHtR and BFM percentage are probably appropriate methods to evaluate obesity in children. Moreover, the WHtR showed a better correlation with blood markers of obesity comorbidities, such as HOMA-IR, lipid profile, and hs-CRP, probably because of its better correlation with fat mass [25, 28]. BFM also showed a greater correlation than BMI z-score with insulin resistance markers and hs-PCR, most probably because it was determined by a direct method of assessment of BFM, as shown by Agbaje A [28].

Regarding SUA levels, the results showed a direct correlation with WHtR but not with BMI z-score or BFM percentage. This could be related to the distribution of fat, with abdominal fat, particularly visceral fat, being more relevant when it concerns to metabolic changes [32] leading to changes in SUA [16, 33]. Moreover, some studies, such as Jørgensen et al. [24], have shown a reduction in SUA after weight loss in obese children, in relation to BMI z-score, but these authors did not use any body composition analysis, and fat mass reduction would probably explain this relationship better, as shown in the paper by Togashi, Masuda, and Iguchi [33]. There was also a correlation between SUA levels and HOMA-IR, ALT, and triglyceride levels, probably in relationship with BFM percentage, in accordance with the studies of Thomazini et al. [13] and Daniali et al. [16]. In the first study, a relationship was found between some comorbidities' markers and the levels of SUA. In Kelishadi's systematic review and meta-analysis, the association between SUA and the main components of pediatric metabolic syndrome was confirmed [16].

Multiple linear regression analysis showed that an increase in WHtR and BFM percentage was positively associated with increasing SUA levels after adjusting for confounding variables (Table 3).

Not all children included in the study underwent abdominal US. However, since the relationship between SUA levels and hepatic steatosis has been described [19, 24, 26], we decided to perform an analysis within this group. Our results revealed that the group with steatosis had more severe obesity, with a higher BMI z-score, WHtR, and greater BFM percentage, as shown before by Sartorio et al. [19] and Mohamed, Jalaludin, and Zaini [20]. SUA levels were significantly higher in the subgroup with steatosis than in the group without steatosis. The relationship between SUA levels and hepatic lesions is well described in the literature [19, 20, 25, 26]. Sartorio et al. [19] and Di Bonito et al. [25] even considered SUA a possible MAFLD predictor. In addition, as described in the literature [19, 20, 25], in our study, the subgroup with steatosis had higher insulin resistance and a worse lipid profile (higher triglycerides and lower HDL). The difference in hs-CRP levels was also statistically significant, something already described in the adult population [19, 26], but contrary to what the study by Sartorio et al. and his team conclude, where there was no statistically significant difference in the pediatric population analyzed [19]. In this subgroup, as in the global analysis, all the three methods of body assessment showed a positive correlation, with WHtR maintaining a better correlation with serologic markers of obesity complications. This better correlation was seen with ALT, HOMA-IR, and hs-CRP. In this subgroup, SUA levels showed a positive correlation with BMI z-score and WHtR, and with more seric markers than in the global study, with correlation with both hepatic enzymes, HOMA-IR, HDL, and triglycerides.

An additional comparison was made between the group with and the group without hepatic study (Supporting Table 1), and the mean SUA levels and hepatic enzymes were not significantly different between the two groups, probably meaning that the same conclusions could be extrapolated to the whole population study.

Therefore, our results support that hyperuricemia was related to insulin resistance markers, lipid profile, and hepatic enzymes, suggesting it can be a good predictor of obesity complications. These outcomes fulfilled our hypothesis by demonstrating that hyperuricemia may be associated with obesity and probably its comorbidities. Increased SUA levels were linked to the severity of obesity, and a stronger correlation was observed with body assessment methods that are more closely related to body fat quantification and distribution. Another interesting result is its relationship with the presence of hepatic steatosis. Since abdominal US only has good sensitivity when fat infiltration is more than 30%, perhaps uric acid could be an earlier indicator of MAFLD.

Some recent studies discussed the possible benefit of the treatment of hyperuricemia, but only in severe cases, with SUA > 9.34 mg/dL [34], where symptoms are usually present in an early age, which is not the case in obesity-related hyperuricemia. Our analysis comprises a significant number of children, with a relatively young mean age (11.3 years old) with almost the same proportions of boys and girls. Another strength of this study is that body measurements were performed using anthropometric (BMI z-score and WHtR) and nonanthropometric methods (bioimpedance), all measurements were made only by two experienced pediatricians, and all USs were made by radiologists specialized in pediatrics.

Although this study was performed in a large number of obese children, it has some limitations. Body composition was not analyzed using DEXA, which is currently the gold standard method. Body composition changes related to growth and puberty were also not well scrutinized making their effects harder to interpret. Blood pressure measurements were not included in the study, a well-described cardiovascular complication related to hyperuricemia.

## 5. Conclusion

This study aimed to determine whether there is an association between hyperuricemia, body composition, and comorbidities in obese children and adolescents. The results showed that all three assessment methods used had a good correlation with each other, with SUA levels and the comorbidities studied. Nevertheless, WHtR and BFM percentage seem to have a better relation with the risk of developing comorbidities than BMI z-score, with WHtR being easy to apply in any clinical practice setting. SUA levels appear to be related to insulin resistance and the grade of inflammatory state of obesity, which could be a potential follow-up marker in obese pediatric population. The association between SUA and MAFLD, a very prevalent comorbidity in obese children, shows a potential predictor of steatosis. Future studies could further clarify the relationship between blood markers (namely hyperuricemia) and body composition in obese children, in order to prevent negative health consequences.

## Figures and Tables

**Table 1:** Descriptive statistics of study population.

	Total (*n* = 505)	No steatosis (*n* = 207)	Steatosis (*n* = 96)	*p* value
Sex—*n* (%)				0.002
Male	261 (51.7)	92 (44.4)	61 (63.5)	
Female	244 (41.3)	115 (55.6)	35 (36.5)	
Age (years)^1^	11.31 ± 3.2	11.36 ± 3.3	12.66 ± 2.8	< 0.001
BMI *z*-score^2^	2.77 (2.46–3.21)	2.68 (2.41–3.04)	2.92 (2.68–3.56)	< 0.001
WtHR^2^	0.60 (0.57–0.64)	0.61 (0.57–0.64)	0.63 (0.60–0.69)	< 0.001
Acanthosis—*n* (%)				
Yes	153 (30.3)			
Fat mass percentage^1^	42.7 ± 6.2	42.6 ± 5.8	45.0 ± 6.0	< 0.001
Muscle mass percentage^1^	30.7 ± 3.6	30.8 ± 3.6	30.1 ± 3.5	0.14
Seric uric acid (2–5.5 mg/dL)^1^	4.9 ± 1.3	4.7 ± 1.2	5.5 ± 1.3	< 0.001
AST (10–30 U/L)^2^	20 (17–25)	20 (17–24)	21 (17–26.8)	0.096
ALT (10–36 U/L)^2^	18 (14–24)	17 (13–21.3)	22.5 (15–39)	< 0.001
Fast glucose (70–105 mg/dL)^2^	85 (80–89)	84 (80–88)	86 (80–90.8)	0.13
HbA1c (4%–6%)^2^	5.3 (5.1–5.5)	5.3 (5.1–5.5)	5.3 (5.1–5.5)	0.66
Insulin (2.6–24.9 μU/mL)^2^	17.7 (12–27.4)	17.6 (12.3–26.1)	26.9 (17.13–37.6)	< 0.001
HOMA-IR^2^	3.69 (2.45–5.81)	3.59 (2.60–5.34)	5.61 (3.54–8.18)	< 0.001
Total cholesterol (0–200 mg/dL)^2^	152 (136–170)	151 (131–170)	149 (137.3–170)	0.894
LDL (< 110 mg/dL)^2^	88 (72–103)	87 (72–103)	86 (72.3–98)	0.687
HDL (> 45 mg/dL)^2^	46 (40–54)	45 (40–53)	42 (37–49)	< 0.001
Triglycerides (< 90 mg/dL)^2^	83 (58–110)	82 (57–105)	98 (72–128.8)	< 0.001
hs-CRP (< 1 mg/L)^2∗^	1.83 (0.83–3.96)	1.77 (0.81–4.06)	2.54 (1.33–4.76)	< 0.001
Abdominal US—*n* (%)				
Yes	303 (60.0)			
Hepatic steatosis—*n* (%)				
Yes	96 (31.7)			

*Note:* (Laboratory reference value, units). Analysis of population group evaluated by abdominal ultrasound.

Abbreviations: hs-CRP = high-sensitive C-reactive protein, US = ultrasound.

^1^Mean ± standard deviation.

^2^Median (interquartile range).

⁣^∗^37 missing values for hs-CRP.

**Table 2:** Bivariate analysis between three nutritional assessment methods and serum uric acid.

	Age	BMI zs	WHtR	BFM %	MM %	SUA	AST	ALT	HbA1c	Insulin	HOMA-IR	TC	LDL	HDL	Trig	hs-CRP
BMI *z*-score	−0.17⁣^∗^	—	0.641⁣^∗^	0.551⁣^∗^	−0.536⁣^∗^	0.054	0.101⁣^∗∗^	0.199⁣^∗^	0.108⁣^∗∗^	0.171⁣^∗^	0.163⁣^∗^	0.101⁣^∗∗^	0.126⁣^∗∗^	−0.144⁣^∗∗^	0.115⁣^∗∗^	0.292⁣^∗^
WHtR	0.21⁣^∗^	0.641⁣^∗^	—	0.588⁣^∗^	−0.494⁣^∗^	0.212⁣^∗^	−0.041	0.238⁣^∗^	0.055	0.363⁣^∗^	0.345⁣^∗^	0.081	0.123⁣^∗∗^	−0.177⁣^∗^	0.183⁣^∗^	0.345⁣^∗^
BFM percentage	0.106⁣^∗∗^	0.551⁣^∗^	0.588⁣^∗^	—	−0.931⁣^∗^	0.01	−0.157⁣^∗^	0.049	0.036	0.276⁣^∗^	0.249⁣^∗^	0.094⁣^∗∗^	0.111⁣^∗∗^	−0.157⁣^∗^	0.118⁣^∗∗^	0.45⁣^∗^
Serum uric acid	0.504⁣^∗^	0.054	0.212⁣^∗^	0.01	0.164⁣^∗^	—	−0.082	0.206⁣^∗^	−0.033	0.384⁣^∗^	0.372⁣^∗^	−0.046	0.012	−0.362⁣^∗^	0.263⁣^∗^	0.059

*Note:* The cells with no ⁣^∗^ or ⁣^∗∗^ are correlations not statistically significant. Correlation coefficients are represented.

Abbreviations: BFM = body fat mass, BMI = body mass index, hs-CRP = high-sensitive C-reactive protein, MM = muscle mass, TC = total cholesterol, Trig = tryglycerides, WHtR = waist-to-height ratio.

⁣^∗^*p* value < 0.001.

⁣^∗∗^*p* value < 0.05.

**Table 3:** Multiple linear regression with serum uric acid levels as dependent variable.

Independent variables	*β* (95% CI)	*p* value
BFM percentage	0.118 (0.048–0.188)	< 0.001
MM percentage	0.269 (0.152–0.387)	< 0.001
Waist-to-height ratio	2.764 (0.973–4.556)	0.003
Age	0.065 (0.016–0.114)	0.01
ALT	0.01 (0.004–0.016)	< 0.001
Triglycerides	0.002 (0.00–0.004)	0.026
HDL	−0.022 ([−0.03]–[−0.014])	< 0.001

*Note:* Relation with body fat mass percentage, with adjustment for confounding variables (muscle mass percentage, waist-to-height ratio, age, ALT, triglycerides, and HDL).

Abbreviations: BFM = body fat mass, MM = muscular mass.

## Data Availability

The data that support the findings of this study are available from the corresponding author, João Vasco, upon reasonable request.

## Nomenclature

ALT: Alanine transaminase
AST: Aspartate transaminase
BFM: Body fat mass
BMI: Body mass index
COSI: Childhood obesity surveillance initiative
DEXA: Dual-energy X-ray absorptiometry
HbA1c: Glycated hemoglobin
HDL: High-density lipoprotein
HOMA-IR: Homeostatic model assessment for insulin resistance
hs-CRP: High-sensitive C-reactive protein
IQR: Interquartile range
LDL: Low-density lipoprotein
MM: Muscular mass
MAFLD: Metabolic dysfunction-associated fatty liver disease
SD: Standard deviation
SUA: Serum uric acid
US: Ultrasound
WHtR: Waist-to-height ratio
WHO: World Health Organization

## Supporting Information

Supporting information is available together with the manuscript. The following supporting tables can be found in this document: Supporting Table 1: Descriptive statistics of study population, comparing the groups with and without hepatic study. Supporting Table 2: Bivariate analysis between three nutritional assessment methods and serum uric acid in the subgroup with hepatic steatosis.
