# Supplementary material for: Association Between Hyperuricemia, Body Composition, and Comorbidities in an Obese Pediatric Population
Source: J Nutr Metab. 2025 Mar 3;2025:2768062. doi: 10.1155/jnme/2768062 (PMC11986183; doi:10.1155/jnme/2768062)
Supplement: Supporting Information — Additional supporting information can be found online in the Supporting Information section. [file 2768062.f1.docx]

**Supplementary table 1.** Descriptive statistics of study population, comparing the groups with and without hepatic study.

|  |  | **Hepatic study**  (n=303) | | **No hepatic study** (n=202) | | **p value** |
| --- | --- | --- | --- | --- | --- | --- |
|  | Sex - n (%) |  |  |  |  | 0.526 |
|  | Male | 153 | (50.5) | 108 | (53.5) |  |
|  | Female | 150 | (49.5) | 94 | (46.5) |  |
|  | Age (years)^1^ | 11.77 ± 3.19 | | 10.62 ± 3.18 | | <0.001 |
|  | BMI z-score^2^ | 2.79 | (2.51-3.23) | 2.75 | (2.42-3.19) | 0.302 |
|  | WtHR^2^ | 0.61 | (0.58-0.65) | 0.59 | (0.57-0.64) | 0.004 |
|  | Fat mass percentage^1^ | 43.2 ± 5.97 | | 41.8± 6.4 | | 0.005 |
|  | Muscle mass percentage^1^ | 30.6 ± 3.6 | | 31 ± 3.7 | | 0.274 |
|  | Seric Uric Acid (mg/dL)^1^ | 4.98 ± 1.29 | | 4.78 ± 1.19 | | 0.076 |
|  | AST (U/L)^2^ | 20 | (17-25) | 21 | (17.8-25) | 0.203 |
|  | ALT (U/L)^2^ | 18 | (14-24) | 18 | (14-23) | 0.366 |
|  | Fast Glucose (mg/dL)^2^ | 85 | (80-89) | 84 | (79-89) | 0.356 |
|  | HbA1c (%)^2^ | 5.3 | (5.1-5.5) | 5.3 | (5.2-5.5) | 0.025 |
|  | Insulin (µU/mL)^2^ | 18.9 | (13.5-29.1) | 15.5 | (10.3-23.7) | <0.001 |
|  | HOMA-IR^2^ | 3.97 | (2.8-6.2) | 3.2 | (2.1-5.1) | <0.001 |
|  | Total Cholesterol (mg/dL)^2^ | 151 | (132-170) | 155 | (138-171) | 0.155 |
|  | LDL (mg/dL)^2^ | 87 | (72-103) | 90 | (72-106) | 0.303 |
|  | HDL (mg/dL)^2^ | 44 | (39-52) | 48 | (41-56) | 0.001 |
|  | Triglycerides (mg/dL)^2^ | 86 | (61-114) | 76 | (56-101) | 0.028 |
|  | hs-CRP (mg/L)^2^ | 2.14 | (0.88-4.43) | 1.53 | (0.73-3.48) | 0.047 |
|  | Footnote: ^1^Mean ± standard deviation; ^2^Median (interquartile range); hs-CRP High sensitive C reactive protein; US - ultrasound; *37 missing values for hsRP | | | | | |
|  |  |  |  |  |  |  |
|  | | | | | | |

**Supplementary table 2.** Bivariate analysis between three nutritional assessment methods and serum uric acid in the subgroup with hepatic steatosis. Correlation coefficients are represented.

|  | Age | BMI zs | WHtR | BFM % | MM % | SUA | AST | ALT | HbA1c | Insulin | HOMA-IR | TC | LDL | HDL | Trig | hs-CRP |  |
| --- | --- | --- | --- | --- | --- | --- | --- | --- | --- | --- | --- | --- | --- | --- | --- | --- | --- |
| BMI z-score | 0.077 | - | 0.783* | 0.669** | -0.579** | 0.211* | 0.022 | 0.202 | 0.147 | 0.394** | 0.381** | 0.005 | 0.005 | -0.08 | 0.09 | 0.483* |  |
| WHtR | 0.273** | 0.783* | - | 0.555** | -0.436** | 0.324* | 0.117 | 0.358** | 0.121 | 0.398** | 0.395** | 0.005 | 0.021 | -0.127 | 0.112 | 0.47** |  |
| BFM percentage | 0.082 | 0.669** | 0.555** | - | -0.94** | -0.132 | -0.201 | 0.038 | 0.025 | 0.239* | 0.194 | -0.027 | -0.018 | 0.155 | -0.072 | 0.657** |  |
| Serum Uric Acid | 0.506** | 0.211* | 0.324** | -0.132 | 0.164* | - | 0.276** | 0.375** | -0.018 | 0.319** | 0.353** | 0.08 | 0.087 | -0.268** | 0.312** | -0.069 |  |
| Footnote: *P value<0.001. **P value<0.05. The cells with no * or ** are correlations not statistically significant. BMI - body mass index; WHtR - waist-to-height ratio; BFM - body fat mass; MM - muscle mass; TC - total cholesterol; Trig - tryglicerydes; hs-CRP - high sensitivity C reactive protein. | | | | | | | | | | | | | | | | |  |
|  |  |  |  |  |  |  |  |  |  |  |  |  |  |  |  |  |  |
